# MsDD: A novel NDN producer mobility support scheme based on multi-satellite data depot

**DOI:** 10.1371/journal.pone.0310379

**Published:** 2024-09-12

**Authors:** Zhiguo Liu, Qing Zou, Lin Wang, Zhengxia Liu

**Affiliations:** 1 Communication and Network Laboratory, Dalian University, Dalian, China; 2 College of Environment and Chemical Engineering, Dalian University, Dalian, China; National Yang Ming Chiao Tung University, TAIWAN

## Abstract

The Named Data Networking (NDN) is currently an important future network framework, and the mobility issue of producers within NDN is a primary challenge. However, in environments characterized by frequent producers mobility, traditional producer mobility support schemes still encounter issues such as excessive consumer delays and interest packet loss. With the development of The 6th generation communication technology (6G), integrating ground networks with satellites has emerged as a potential solution to address the aforementioned problems. In this paper, we propose an NDN producer mobility support scheme based on multi-satellite data depot, named MsDD. The proposed scheme proactively caches producer data into a data depot built on a low-earth orbit satellite constellation to minimize the impact of NDN producer mobility on network performance. We design data depot construction strategy, in-network caching strategy, and routing strategy based on forwarding hint to facilitate effective communication in satellite networks. Experimental results using ndnSIM demonstrate that compared with other existing schemes, MsDD can effectively shield the impact of producer mobility on consumer delay, delivery ratio, and signaling overhead, and MsDD has a clear advantage in terms of consumer delay and delivery ratio.

## 1 Introduction

With the rapid development of network communication technology and hardware, the connectivity provided by the Internet and its low storage costs have made a vast amount of new content accessible. As a result, the volume of data on the network is growing at an astonishing rate. Statistics show that in 2008, the amount of information on the Internet reached 500 exabytes, surpassing a zettabyte in 2010, growing to 1.8ZB in 2011, and reaching 44ZB in 2020 [1]. Consequently, network users are increasingly focusing on the content itself rather than just its storage location. Additionally, the growth rate of global mobile data traffic is nearly twice that of fixed IP traffic, and consumer Video-on-Demand (VoD) traffic is expected to increase by nearly double, indicating that mobile multimedia communication will gradually become mainstream. With the explosive growth in content volume and the rise of video-on-demand and live streaming, future network architectures will have higher requirements for bandwidth, latency, and other aspects of content transmission [2].

Named Data Networking (NDN) is one of the future network architectures that meets these higher requirements [3]. Unlike traditional IP networks, NDN is content-centric rather than host-centric, placing content at the center of network communication rather than relying solely on host addresses [4]. NDN, as a popular new generation network architecture in recent years, has many unique advantages and potentials. At the same time, it also brings corresponding challenges for researchers, such as mobility support [5]. Mobility support has been a hot topic in NDN research, involving both consumer and producer mobility issues [6]. Due to NDN’s inherent support for consumer mobility through the retransmission mechanism of interest, the current focus of NDN mobility support research is on supporting producer mobility [7].

Most existing NDN producer mobility support schemes use reactive techniques to restore the network after producer mobility. Therefore, it is crucial to utilize caching-based schemes by moving the produced data to an easily accessible location to support seamless producer mobility [8]. Currently, caching-based schemes use ground routers and other devices as cache points or aggregation points for content data. However, with the rise of 6G communication technology and advancements in satellite communication hardware, supporting seamless producer mobility in an integrated space-ground environment has become achievable [9]. Therefore, in this paper, to minimize the impact of producer mobility on the NDN network and ensure communication quality, we propose an NDN producer mobility support scheme based on a multi-satellite data depot called MsDD. MsDD utilizes the characteristics of low orbit satellites such as low latency, strong signal, low cost, wide coverage, and easy access by ground devices to form a distributed data depot with multiple low orbit satellite nodes. The data packets generated by producers can be cached in this data depot, allowing interest packets and data packets to aggregate within the depot to maintain communication between consumers and mobile producers. Specifically, the main contributions of this paper are as follows:

- We construct a multi-satellite data depot model consisting of a Walker constellation and GEO(Geostationary Orbit) satellites. This data depot aggregate interest packets and data packets within the depot to effectively shield the impact of producer mobility on network performance.
- We design a forwarding-hint-based routing strategy that takes into account the unique attributes of the Walker constellation. This routing strategy ensures the effective transmission of data packets and interest packets within the satellite network.
- We design a probability-based in-network caching strategy for MsDD. This strategy caches data packets with different probabilities based on the popularity of the data packets and the priority of the satellite nodes. It reduces cache redundancy and decreases the retrieval time of interest packets within the data depot.

The remaining sections of this paper are organized as follows. Section 2 provides an overview of the relevant background technologies of NDN and related work on producer mobility support. Section 3 introduces the proposed MsDD producer mobility support scheme. Section 4 explains the results of the simulation experiments. Finally, in section 5, we summarize our work.

## 2 Background and related work

Named Data Networking (NDN), as an implementation architecture of Information-Centric Networking (ICN), is a highly promising future network architecture. In NDN, there are mainly two types of packet transmissions: Interest packets and Data packets, which form the basis for all communications [10]. In NDN, the requester of content is called the consumer, and the provider of content is called the producer. Interest packets and data packets carry a common name indicating the content required for that communication. Consumers request content by sending interest packets to producers, and these interest packets are routed based on the longest prefix match of the content name, using the Forwarding Information Base (FIB) to guide them towards the producers. When producers receive interest packets, they respond with data packets containing the requested content [11].

In NDN, although the content name is decoupled from its location, however, the content name is not separated from its location [11]. When producers move, interest packets may still be routed to their old locations, resulting in packet loss as the desired content cannot be retrieved [12]. After the event of packet loss, relying solely on interest packet retransmission mechanisms to restore communication is not feasible in large-scale networks. Therefore, designing a more reliable NDN producer mobility support scheme is a crucial topic in current NDN research.

Article [13–15] proposed the anchor-based approach, which are designed based on the Mobile IP protocol [16] used in the current Internet. Article [13] proposed a classic scheme: KITE, which allocates a non mobile anchor point for all mobile nodes to support the mobility of producers, but the anchor point may cause problems such as single point of failure and long forwarding path. The scheme proposed in article [14] uses intermediate content router (CR) and home domain content router (CRH) to build a home domain router, and transmits interest and data packets through tunnels. But like mobile IPv6, the home domain router tunnel will use encapsulation and deencapsulation, which increases the interest retransmission rate and packet drop rate. The scheme proposed in article [15] uses the anchor node to forward interest packets, and uses an update packet to update the prefix generation information of the anchor node. Compared with KITE [13], this scheme shows better performance and reduces retransmission of interest packets. However, the proposed scheme does not determine the selection method of anchor nodes, and produces problem such as inefficient data forwarding path.

Scheme [17] enhances seamless mobility support in NDN by modifying FIB and NDN packets that access routers. Its advantage is that it can provide interrupted content and reduce handoff latency, but its communication overhead in the wide area network is high, and it increases the distance and hops of communication, which is easy to cause the loss and retransmission of interest packets. Scheme [18] uses a naming server to track the location of producers. The naming server facilitates the communication between producers and consumers to a certain extent, but in the environment where producers move frequently, the naming server may provide outdated locations, which may lead to the loss of interest packets and interest retransmission. The article [19] proposed an anchorless mobility support scheme named MAP-ME, which supports real-time communication during producer mobility by dynamically updating the FIB tables of minimal routers in the network. To address packet loss caused by FIB update delays, the scheme includes an additional protocol named “Notification/Discovery”. The most notable drawback of MAP-ME is the triangular routing problem, which can result in unnecessary delays.

For most caching-based schemes [20–23], they employ various methods and techniques to mitigate the impact of producer mobility. The article [20] designed a scheme named PNPCCN, where the producer caches requested content to neighboring routers based on popularity and rarity before moving. This allows consumers to effortlessly retrieve these contents from the neighboring routers. However, this scheme only supports specific content during producer mobility, and requests for unpopular content might be lost. The scheme proposed in article [21] not only pushes content packets but also maintains content availability by placing copies of the data packets, which can lead to significant unnecessary overhead due to excessive redundant copies. Article [22] introduced a scheme named T-Move, which supports producer mobility by caching selected content on an edge router within the network. This scheme enhances router functionality by adding content names, trends, and frequency. Additionally, it introduces control messages GETT (GET Trendiness) and REPT (REPort Trendiness) to obtain recent router information. However, T-Move requires broadcasting these messages to update FIB and cache messages, which inevitably increases signaling overhead during handovers. Article [23] designed a proactive caching scheme based on predictive techniques, utilizing location prediction and user access patterns to proactively cache potential data in real-time at an optimal location near the consumer. This allows consumers to retrieve the required content without their interest packets reaching the mobile producer, thereby avoiding unnecessary delays. Nonetheless, this scheme generates additional signaling and computational overhead, and the unpopular content requests lead to loss and invoke interest retransmission issue.

Caching-based producer mobility support schemes need to prioritize the design of content caching strategies. Our proposed MsDD focuses primarily on in-network caching schemes. LCE (Leave Copy Everywhere) [24] is the default in-network caching scheme in NDN, which allows routers to cache all incoming data packets. However, this leads to high cache redundancy and low cache utilization. LCD (Leave Copy Down) [25], originally proposed for hierarchical web caching systems, only caches content at the next hop of the current serving node along the path to the requester whenever content requests are served from the cache or content source. In Prob [26], cache nodes decide whether to cache incoming data based on a certain probability. ProbCache [27] is a dynamic probabilistic caching mechanism that calculates the caching probability based on the cache capacity of remaining routers along the transmission path and the hop count from the current cache node to the server. Betw/EgoBetw [28] considers the centrality of cache nodes. However, this strategy has high complexity and requires global node information and communication overhead between nodes before network operation. CCS/CES [29] is a lightweight and reactive NDN-compliant caching scheme that applies two different caching strategies by dividing the network into edge and core segments. These strategies collectively consider content popularity and freshness, and do not require global node information. The PTF proposed in article [30] caches content by calculating cache benefit, which comprehensively considers the content popularity, cache location, and content freshness, and PTF predicts the cache benefit of new content by using Grey Model.

## 3 Multi-satellite data depot

**Fig 1:**
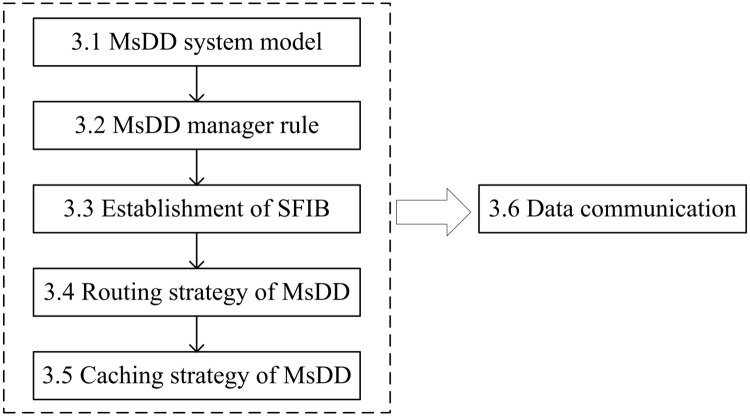
The structure of this section.

In this section, we will systematically introduce how MsDD operates to address the mobility issues of NDN producers. The structure of this section is illustrated in Fig 1.

### 3.1 The model of MsDD

**Fig 2:**
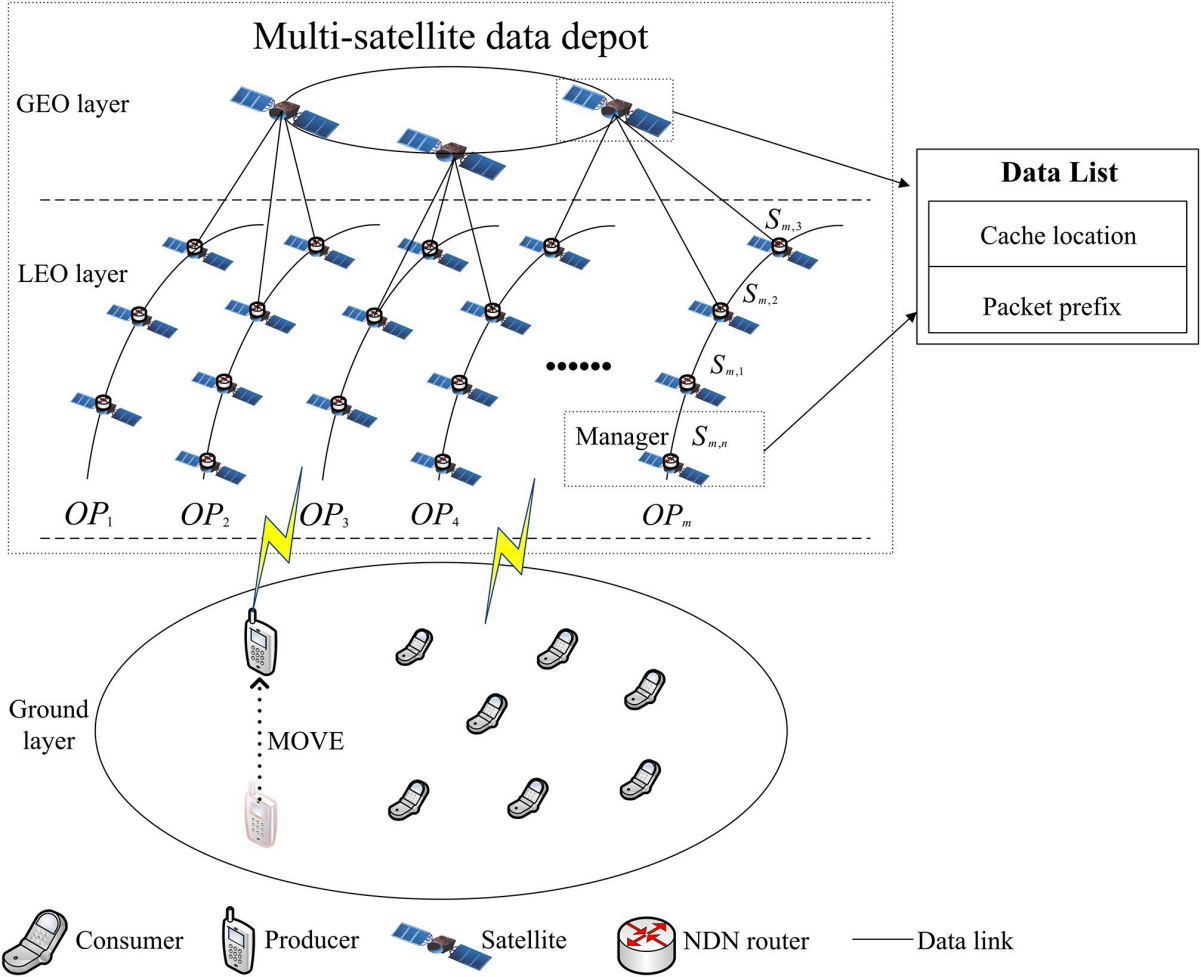
The model of MsDD.

The MsDD model consists of three layers: the ground layer, the LEO (Low Earth Orbit) layer, and the GEO (Geostationary Orbit) layer, as illustrated in Fig 2. In ground layer, consumers and mobile producers within the coverage of satellites can directly communicate with LEO layer satellites by using hardware devices. The LEO and GEO layers collectively form a distributed data depot. The LEO layer comprises *m* ordered polar orbit planes, forming a Walker constellation, with each plane uniformly distributing *n* ordered LEO satellites. Each LEO satellite is uniquely defined by the prefix /*sat*/*OP*_*h*_/*SP*_*i*_, where /*sat* denotes it as a satellite node, /*SP*_*i*_ denotes its orbit, and /*SP*_*i*_ denotes its sequence in the orbit, thus there exists a set of low-earth orbit satellite nodes denoted as *S* = {*S*_*h*,*i*_, *h* = 1, 2, ⋯, *m*, *i* = 1, 2, ⋯, *n*}. The GEO layer consists of three GEO satellites evenly distributed at intervals above the equator, forming a GEO constellation whose orbital plane coincides with the equatorial plane. These three GEO satellites completely cover the entire LEO layer and are responsible for sending update information to the LEO satellites.

In addition to the FIB (Forwarding Information Base), PIT (Pending Interest Table), and CS (Content Store) tables inherent to NDN nodes, we introduce a Data List table, denoted as DL. Within each orbit, several LEO satellites will be selected as managers according to MsDD manager rules. Each manager and three GEO satellites carry a same DL. Each DL entry comprises the location (node prefix) where data packets are first cached and the prefix of the data packet name.

### 3.2 Manager rule of MsDD

**Fig 3:**
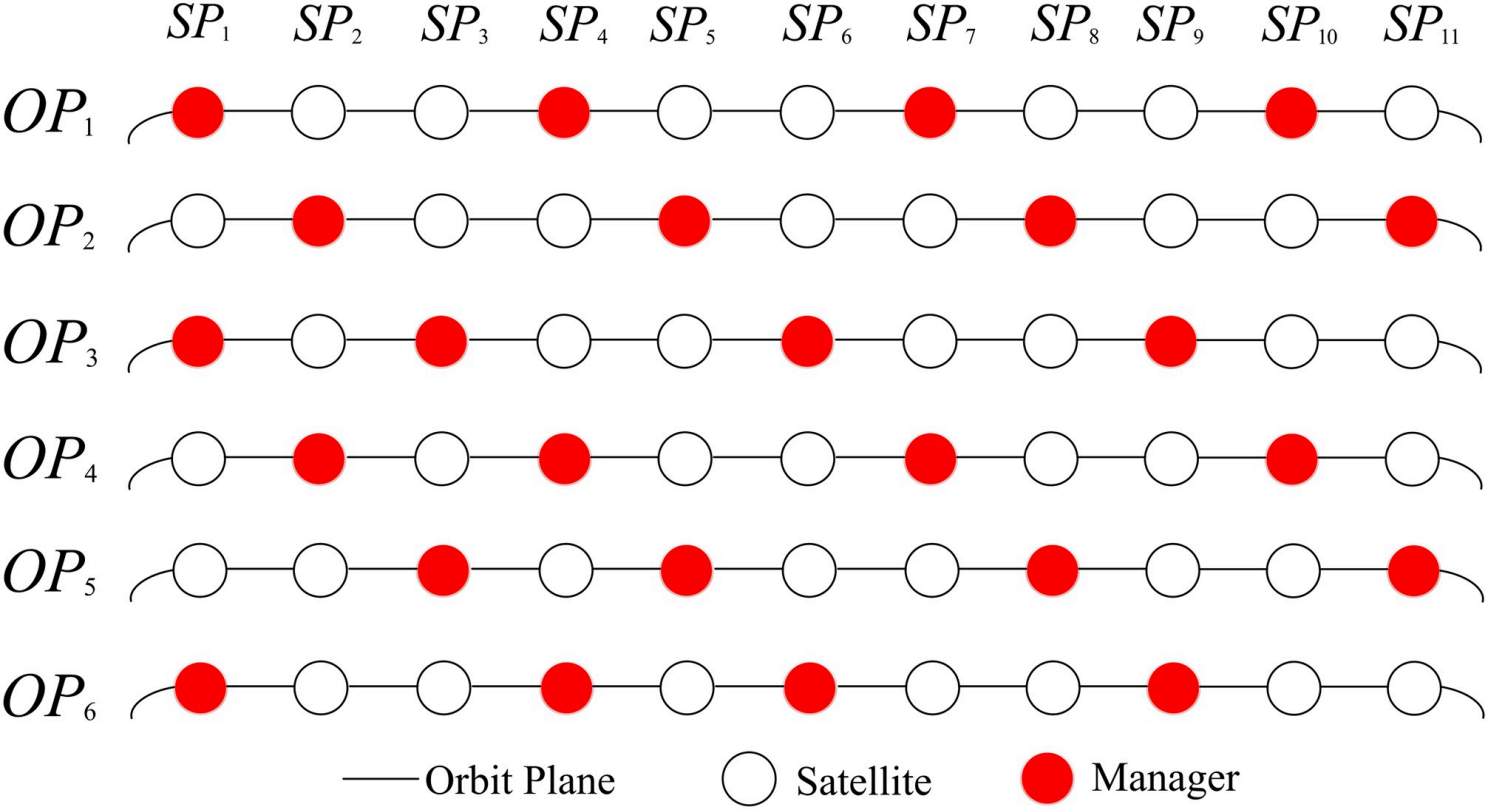
Arrangement of managers in the Iridium constellation when M = 4.

In MsDD, we specify that each orbit is managed by *M* managers, spaced at intervals of $\lceil \frac{n}{M}\rceil -1$ nodes within the orbit. Additionally, in MsDD, we define that if a manager on orbit /*OP*_*h*_ is *S*_*h*,*i*_, then the manager on the adjacent orbit /*OP*_*h*+1_ is *S*_*h* + 1, *i*+1_, and this pattern continues for other orbits. This arrangement method ensures a roughly equal number of managers across different latitudes. Fig 3 illustrates the arrangement scenario for an Iridium constellation (*n* = 11, *m* = 6) when *M* = 4.

### 3.3 Establishment of SFIB in MsDD

In MsDD, in addition to the FIB carried by ground devices and LEO satellites, LEO satellites also maintain an SFIB (Satellite FIB). The distinction between FIB and SFIB in MsDD is as follows:

- FIB: Responsible for satellites to ground communication. FIB entries on LEO satellites include different faces defined by frequency bands in the downlink.
- SFIB: Responsible for inter-satellite communication. An SFIB entry includes the definition of a satellite prefix, faces, and manager identifiers.

The FIB is inherent to NDN nodes, so we mainly explain the process of establishing SFIB in this section. The establishment of SFIB is crucial preparation for data communication. It is essential to note that when the constellation structure is determined, the completed SFIB entries should not be limited by lifetime and deleted. Therefore, SFIB entries are not subject to lifespan considerations once established.

In constructing SFIB entries for satellites on the same orbit in MsDD, the process follows these steps:

Step 1: Satellite *S*_*h*,*i*_ sends a Pub-A message from each of its two relay faces communicating with other satellites on the same orbit. This Pub-A message includes prefix information, hop count, and manager identifiers of *S*_*h*,*i*_.
Step 2: Face *f*_*a*_ of Satellite node *S*_*h*,*i*^+^_ receives the Pub-A message sent by *S*_*h*,*i*_ and processes it as follows:

- If an SFIB entry with prefix /*sat*/*OP*_*h*_/*SP*_*i*_ exists in node *S*_*h*,*i*^+^_ and the hop count of Pub-A is lower than the existing one, update the SFIB entry.
- If an SFIB entry with prefix /*sat*/*OP*_*h*_/*SP*_*i*_ exists in node *S*_*h*,*i*^+^_ but the hop count of Pub-A is not lower than the existing one, then no adjustment is made.
- If no SFIB entry with prefix /*sat*/*OP*_*h*_/*SP*_*i*_ exists in node *S*_*h*,*i*^+^_, *S*_*h*,*i*^+^_ creates a new SFIB entry with a prefix of /*sat*/*OP*_*h*_/*SP*_*i*_, face of *f*_*a*_, and records its manager identifier.

Step 3: End of processing.

Since the sequence of satellites on the same orbit remains unchanged, once SFIB entries for satellites on the same orbit are established, there is no need for nodes to resend Pub-A messages to establish these entries again.

Due to the characteristics of polar orbit satellite constellations, when a satellite passes through the North and South Poles, its adjacent orbits will undergo left-right substitution, so it is necessary to dynamically adjust the SFIB table entries for different orbits. MsDD constructs SFIB table entries for different orbits according to the following steps:

Step 1: Satellite *S*_*h*,*i*_ sends a Pub-B message from each of its two relay faces communicating with satellites on different orbit at regular time interval *τ*. *τ* is determined by the characteristics of different satellite constellations. This Pub-B message includes prefix information of *S*_*h*,*i*_.
Step 2: Face *f*_*b*_ of Satellite node $S_{h^{+},i^{+}}$ receives the Pub-B message sent by *S*_*h*,*i*_ and processes it as follows:

- If an SFIB entry with prefix /*sat*/*OP*_*h*_ exists in node $S_{h^{+},i^{+}}$ but face is not *f*_*b*_, update the SFIB entry.
- If an SFIB entry with prefix /*sat*/*OP*_*h*_ exists in node $S_{h^{+},i^{+}}$ and face is *f*_*b*_, then no adjustment is made.
- If no SFIB entry with prefix /*sat*/*OP*_*h*_ exists in node $S_{h^{+},i^{+}}$, $S_{h^{+},i^{+}}$ creates a new SFIB entry with a prefix of /*sat*/*OP*_*h*_/*SP*_*i*_ and face of *f*_*b*_.

Step 3: End of processing.

**Fig 4:**
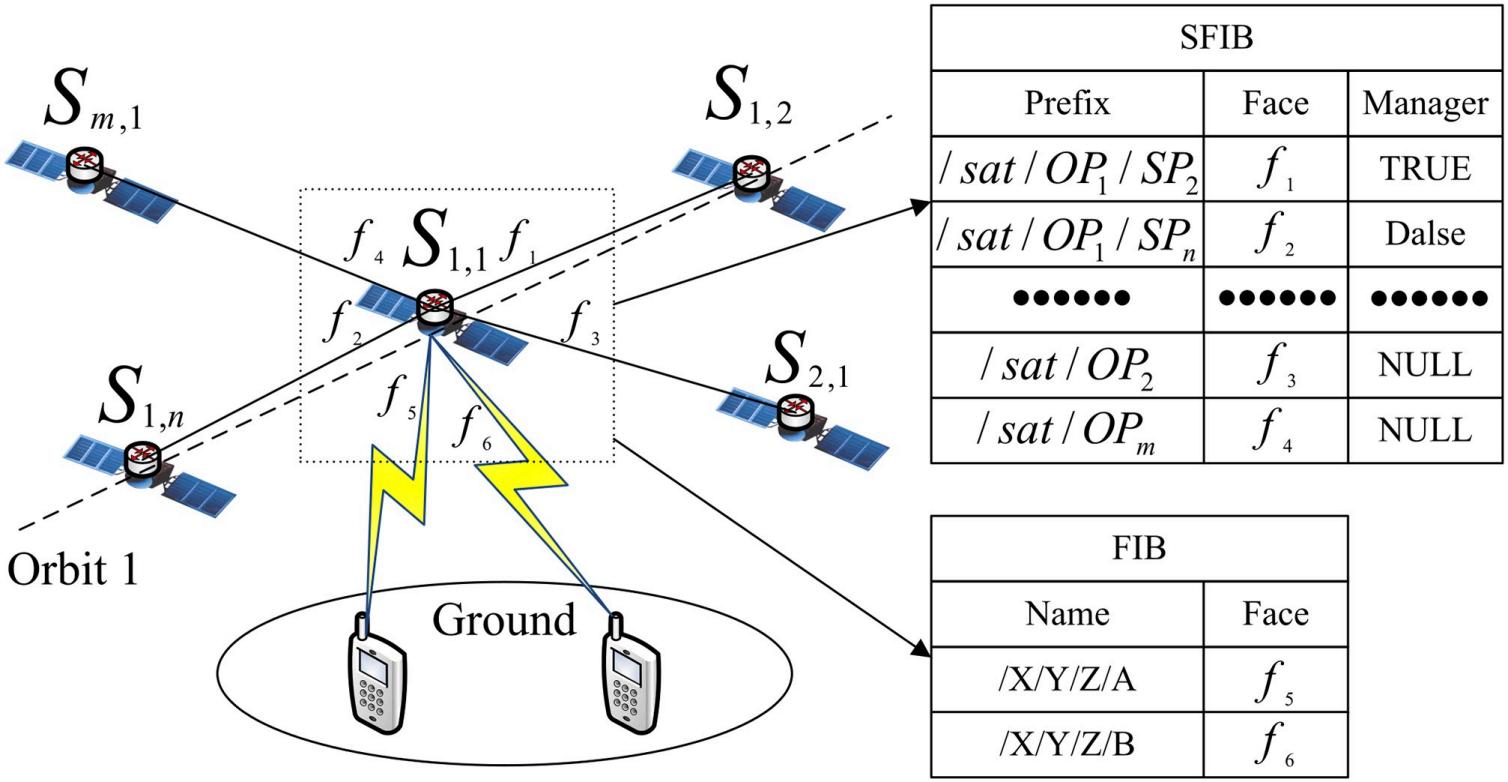
SFIB establishment of satellite *S*_1,1_.

By completing these steps, each LEO satellite node in MsDD creates an SFIB. Each satellite node can utilize its SFIB to communicate with other satellites in the LEO layer. Fig 4 illustrates the situation after satellite *S*_1,1_ completes the construction of its SFIB.

### 3.4 Routing strategy of MsDD

Due to the dynamic characteristics of satellite constellations, traditional NDN routing strategies are not applicable in satellite constellations. Our idea is to first forward the interest packet to the orbit where the destination node is located, and then forward it within the orbit, in order to reduce the impact of the instability of inter satellite links between different orbits on the transmission path. To achieve this goal, we use forwarding hint. The forwarding hint is a locator carried in the interest packet, indicating where to forward the interest packet. By forwarding hint, the core network of NDN can only announce its location in the form of a prefix, which is more scalable than announcing data name prefix [31]. Since we have set a unique prefix name for each satellite in the LEO layer when constructing it, we use the prefix of the destination node as a forwarding hint to route interest packets.

Since ordinary nodes do not carry DL, it is necessary to obtain the prefix name of the target node through the DL carried by the manager. Therefore, ordinary nodes need to first forward interest packets to the nearest manager on the same orbit through FIB entries, and then the manager adds forwarding hint for the interest packets. After receiving a forwarding hint, the interest packet is routed by the manager according to Algorithm 1.

**Algorithm 1** Routing algorithm for interest packet *D*_*int*_.

**Require:**

 /*sat*/*OP*_*h*_/*SP*_*i*_: *prefix* *that* *defines* *node* *S*_*h*,*i*_

 /$/sat/OP_{h^{+}}/SP_{i^{+}}$: *prefix* *that* *defines* *the* *destination* *node* $S_{h^{+},i^{+}}$, *this* *prefix* *as* *the* *forwarding* *hint* *of* *D*_*int*_

 /*sat*/*OP*_0_: *regard* *it* *as* /*sat*/*OP*_*m*_

 *D*: *data* *packet* *that* *requested* *by* *D*_*int*_

1:  **for** *D*_*int*_ forwards to new node *S*_*h*,*i*_ **do**

2:   **if** *D* has already been cached in CS of *S*_*h*,*i*_ **then**

3:    finish forwarding

4:   **end if**

5:   **if** there is a table entry named *D* in the PIT of *S*_*h*,*i*_ **then**

6:    finish forwarding

7:   **end if**

8:   **if** *h* == *h*^+^ **then**

9:    **if** *i* == *i*^+^ **then**

10:     finish forwarding

11:    **else**

12:     forward *D*_*int*_ to new node according to SFIB entries of $S_{h^{+},i^{+}}$

13:    **end if**

14:   **else if** *h* < *h*^+^ **then**

15:    **if** $|h-h^{+}|>\frac{m}{2}$

16:     forward *D*_*int*_ to a new node defined by prefix /*sat*/*OP*_*h*−1_

17:    **else**

18:     forward *D*_*int*_ to a new node defined by prefix $/sat/OP_{(h+1)\;\text{mod}\;m}$

19:    **end if**

20:   **else**

21:    **if** $|h-h^{+}|>\frac{m}{2}$

22:     forward *D*_*int*_ to a new node defined by prefix $/sat/OP_{(h+1)\;\text{mod}\;m}$

23:    **else**

24:     forward *D*_*int*_ to a new node defined by prefix /*sat*/*OP*_*h*−1_

25:    **end if**

26:   **end if**

27: **end for**

### 3.5 Caching strategy of MsDD

Due to the fact that the data depot of MsDD is composed of multiple LEO satellites, it is necessary to design an in-network caching strategy to enhance cache hit rates, reduce cache redundancy, and reduce data retrieval delay.

The in-network caching strategy specifies which satellite nodes along the reverse path should cache the data packet when it returns to the consumer. Our goal is to incorporate content popularity and node priority into mathematical formulas to calculate the probability of caching the data packet at a given node. This aims to reduce the retrieval time of interests in the data depot and improve cache hit rates. The manager rules and routing strategy of MsDD facilitate achieving this objective because interests are routed through manager during each forwarding, leading to a concentration of interests at these nodes. Consequently, we conclude that management nodes have the highest priority, and nodes closer to management nodes have higher priority. Therefore, we have devised the following design for MsDD:

- Introducing a TLV element named *ISLhop* into the interest packet, responsible for recording the number of hops the interest packet has been forwarded between different orbits. When the interest packet is forwarded within the same orbit, *ISLhop* = 0.
- The node is responsible for recording and updating the *ISLhop* of Interest packets received from the face that communicates with satellites in two different orbits.

Based on these conclusions and designs, we define the probability *P*_(*h*, *i*), *D*_ of caching a data packet *D* at a LEO satellite node *S*_*h*,*i*_ as:

$$\begin{array}{c} P_{(h,i),D}=P_{(h,i),D}^{dif}+P_{(h,i),D}^{same}-P_{(h,i),D}^{dif}\cdot P_{(h,i),D}^{same} \end{array}$$ (1)

$$\begin{array}{c} P_{(h,i),D}^{dif}=\{\begin{array}{cc} P_{D}\cdot \frac{2\varepsilon }{\sqrt{2\pi }}\int_{ISLhop_{h,i}-1}^{ISLhop_{h,i}}e^{\frac{{\left(\varepsilon \cdot x\right)}^{2}}{2}}dx & ISLhop_{h,i}\in \left[1,\left\lceil \frac{m}{2}\right\rceil \right]\\ 0 & ISLhop_{h,i}=0 \end{array} \end{array}$$ (2)

$$\begin{array}{c} P_{(h,i),D}^{same}=\{\begin{array}{cc} P_{D}\cdot \frac{2\varepsilon }{\sqrt{2\pi }}\int_{hop_{h,i}-1}^{hop_{h,i}}e^{\frac{{\left(\varepsilon \cdot x\right)}^{2}}{2}}dx & hop_{h,i}=1,2,3,\cdots ,hop_{max}\\ P_{D} & hop_{h,i}=0 \end{array} \end{array}$$ (3)

Where, $P_{(h,i),D}^{dif}$ is the probability of *S*_*h*,*i*_ caching data packet *D* when the data packet is forwarded through different orbits, $P_{(h,i),D}^{same}$ is the probability of *S*_*h*,*i*_ caching data packet *D* when the data packet is forwarded through the same orbit. *P*_*D*_ is the probability of packet *D* being cached at any node, and our scheme uses the method proposed in reference [29] to calculate the value. Through this method, the content popularity and freshness of *D* can be jointly calculated to obtain the value of *P*_*D*_. *ε* is reduce weight and *ε* ∈ (0, 1). The larger the value of *ε*, the greater the trend of *P*_(*h*, *i*), *D*_ decrease.*ISLhop*_*h*,*i*_ is the *ISLhop* of *S*_*h*,*i*_, *hop*_*max*_ is the maximum number of hops from a regular node to the manager within the same orbit, and *hop*_*h*,*i*_ is the number of hops between *S*_*h*,*i*_ and the nearest manager within the same orbit. We assume that after querying the SFIB, *S*_*h*,*i*_ identifies its nearest manager as *S*_*h*,*j*_. Thus, *hop*_*max*_, *hop*_*h*,*i*_ and *ISLhop*_*h*,*i*_ can be calculated as follows:

$$\begin{array}{c} hop_{max}=\left\lceil \frac{n-M}{2M}\right\rceil \end{array}$$ (4)

$$\begin{array}{c} hop_{h,i}=\{\begin{array}{cc} \left|j-i\right| & \left|j-i\right|\leq hop_{max}\\ n-|j-i| & |j-i|>hop_{max} \end{array} \end{array}$$ (5)

$$\begin{array}{c} ISLhop_{h,i}=\frac{ISLhop_{\left(h,i\right),f_{a}}+ISLhop_{\left(h,i\right),f_{b}}}{2} \end{array}$$ (6)

Where, $ISLhop_{\left(h,i\right),f_{a}}$ is the current *ISLhop* value of face *f*_*a*_ recorded by node *S*_*h*,*i*_, $ISLhop_{\left(h,i\right),f_{b}}$ is the current *ISLhop* value of face *f*_*b*_ recorded by node *S*_*h*,*i*_, *f*_*a*_ and *f*_*b*_ are two relay faces communicating with satellites on different orbit.

When a node caches data packets but the Cache Store is full, cache replacement is necessary. We adopt the Least Recently Used (LRU) as the cache replacement strategy. Although it is very simple, it can ensure good performance [32].

### 3.6 Communication process of MsDD

**Fig 5:**
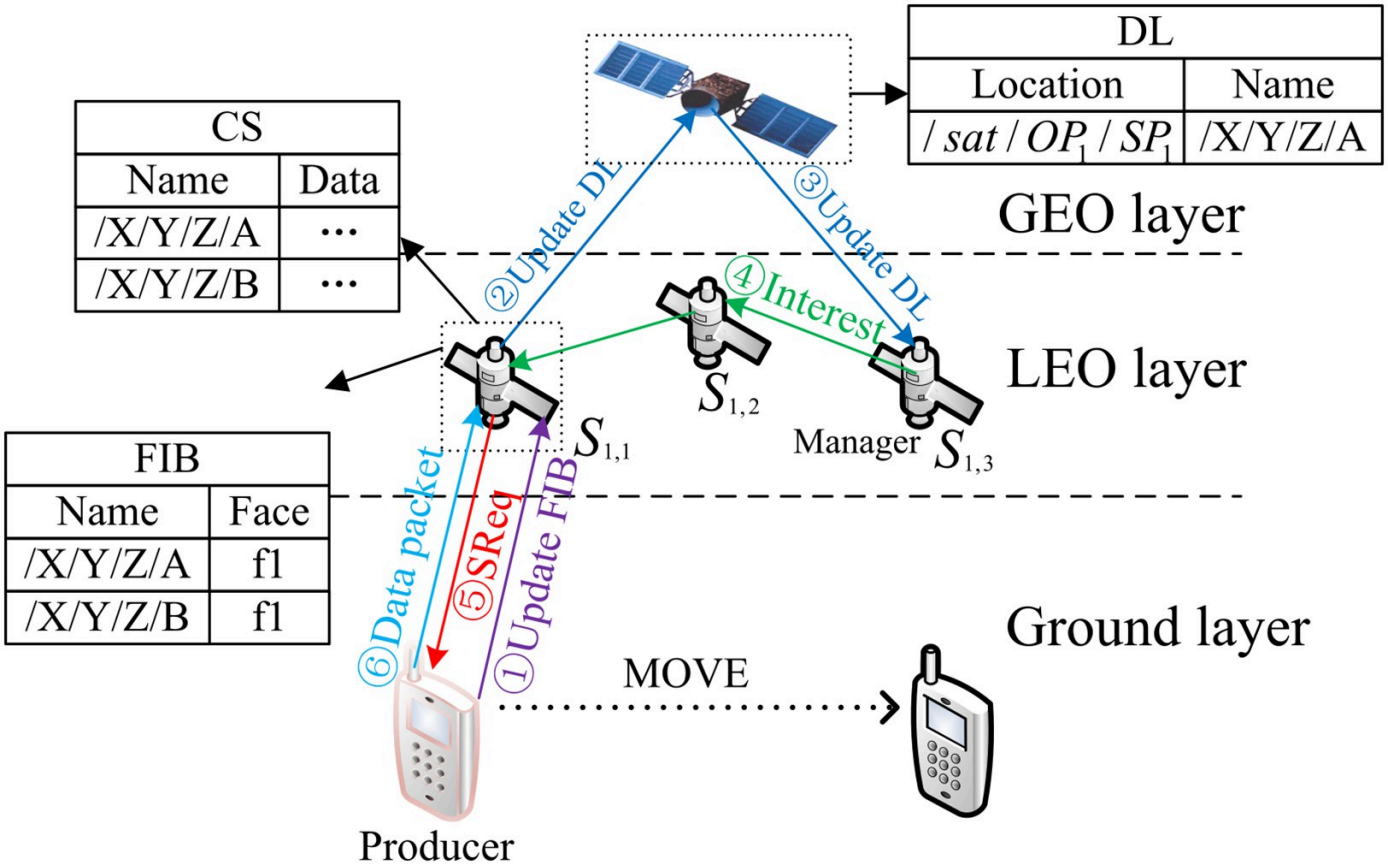
The collection of data packets.

This section will elucidate how interest packets request data packets for data communication within the MsDD framework. Initially, LEO satellite which covers the producer need to collect data packets from producer. This process is driven by a request-based “PULL” method, which does not contravene the fundamental paradigm of NDN. As depicted in Fig 5, the satellite *S*_*h*,*i*_ will undertake the following actions to collect data packet *D* from the ground producer *P* within its coverage:

Step 1: When the mobile producer *P* in ground layer hands over to a satellite *S*_*h*,*i*_, *P* sends a *Collect* message to update the FIB of *S*_*h*,*i*_. The *Collect* message contains the name prefixes of the data objects that *P* possesses.
Step 2: *S*_*h*,*i*_ then sends an update message to the GEO controller. The GEO controller performs a global update, which is responsible for updating the DL, including the prefix of *S*_*h*,*i*_ and the name prefixes of the data objects possessed by *P*.
Step 3: When *S*_*h*,*i*_ receives the interest packet that requests data packet *D*, *S*_*h*,*i*_ sends an *SReq* request for the data packet *D* to *P* according to FIB. After receiving *D*, *S*_*h*,*i*_ caches it.
Step 4: End of processing.

When a consumer from the ground forwards an interest packet *D*_*int*_ requesting the data packet *D* to the LEO satellite $S_{h^{+},i^{+}}$, the data packet retrieval process in MsDD begins:

Step 1: $S_{h^{+},i^{+}}$ forwards the *D*_*int*_ to the nearest manager $S_{h^{+},j}$ according to the SFIB entries.
Step 2: $S_{h^{+},j}$ checks DL table entries and takes the following actions:

- If there is an entry in the DL with the name of *D*, and the cached location is *S*_*h*,*i*_, proceed to Step 3.
- If there is no entry in the DL with the name of *D*, the *D*_*int*_ will wait at $S_{h^{+},j}$ and repeat Step 2.

Step 3: The *D*_*int*_ uses the prefix of *S*_*h*,*i*_ as the forwarding hint, and then $S_{h^{+},j}$ forwards the *D*_*int*_ according to Algorithm 1.
Step 4: The *D*_*int*_ attempts to hit *D* in the cache. If the cache miss occurs at *S*_*h*,*i*_, *S*_*h*,*i*_ will start collecting *D*.
Step 5: End of processing.

**Fig 6:**
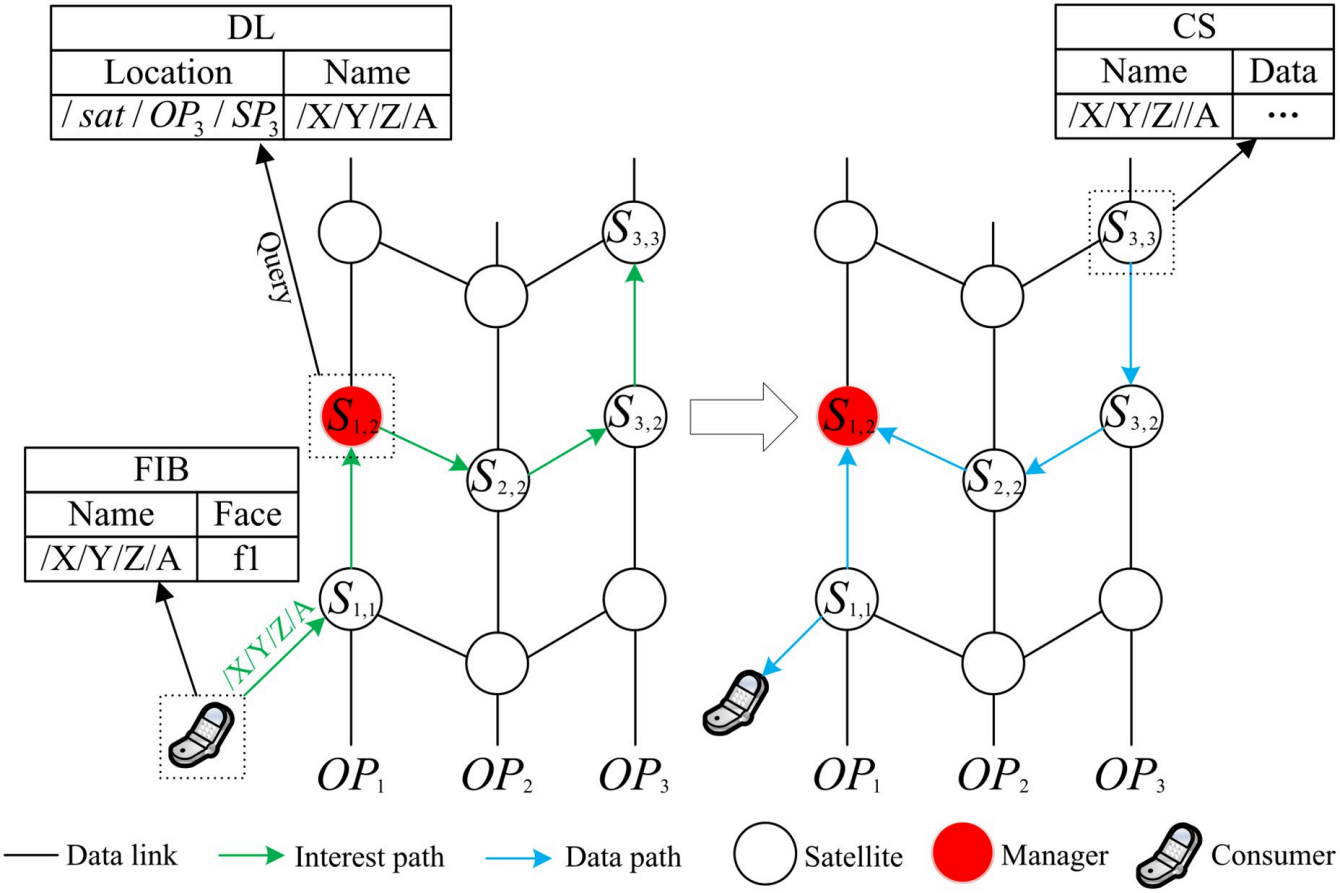
The retrieval of data packets.

The data packet retrieval process is illustrated in Fig 6. The left side of Fig 6 shows the process of sending the interest packet, while the right side shows the process of returning data packet.

**Fig 7:**
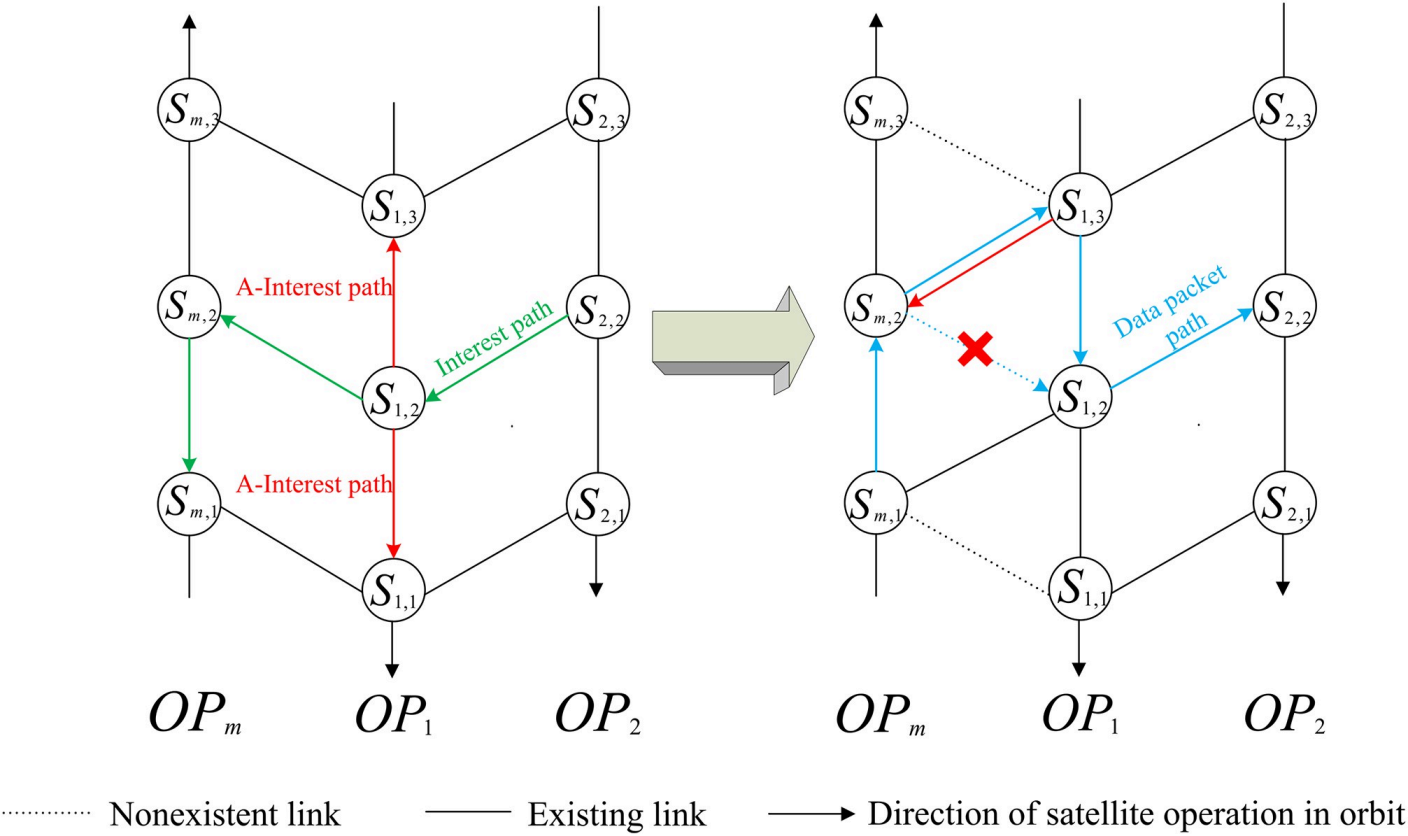
The workflow of A-interest packet in cross-seam.

To prevent packet loss due to link switching at the cross-seam of Walker constellation in the reverse path of data packets, MsDD proposed the following methods to address this issue: When a satellite node needs to send an interest packet through the inter-satellite link of the cross-seam, the satellite node will send an Auxiliary Interest packet (A-Interest) to its neighboring satellite nodes in the same orbit. The difference between the A-interest and the ordinary interest packet is that the hop limit of the A-interest is 2. The process of sending the A-Interest packet is described in Algorithm 2. The purpose of sending the A-Interest packet is to reconstruct a reverse path for the data packet. Fig 7 illustrates this process, showing that when a link switch occurs, a connection is reestablished between *S*_*m*,2_ and *S*_1,3_, and a reverse path of *S*_*m*,2_ → *S*_1,3_ → *S*_1,2_ is constructed.

**Algorithm 2** Routing algorithm for A-Interest packet *D*_*A*−*int*_.

**Require:**

 *RemHops*: *the remaining hops of the A* − *Interest packet*, *with an initial value of* 2

 *D*: *data packet that requested by D*_*A*−*int*_

1:  **for** a node *S*_*h*,*i*_ create or receive a A-interest packet *D*_*A*−*int*_ **do**

2:   **if** *D* has already been cached in CS of *S*_*h*,*i*_ **then**

3:     finish forwarding

4:    **end if**

5:    **if** there is a table entry named *D* in the PIT of *S*_*h*,*i*_ **then**

6:     finish forwarding

7:    **end if**

8:    **if** *RemHops* == 0 **then**

9:     finish forwarding

10:    **else if** *RemHops* == 2 **then**

11:     forward *D*_*A*−*int*_ to two adjacent nodes on the same orbit according to SFIB entries of *S*_*h*,*i*+1_ and *S*_*h*,*i*−1_

12:     *RemHops*−−

13:   **else**

14:    **while** *S*_*h*,*i*+1_ or *S*_*h*,*i*−1_ reestablish a new link with $S_{h^{+},i^{+}}$ **do**

15:     forward *D*_*A*−*int*_ to $S_{h^{+},i^{+}}$

16:     *RemHops*−−

17:    **end while**

18:   **end if**

19:  **end for**

## 4 Simulation and analysis

### 4.1 Simulation environment

**Fig 8:**
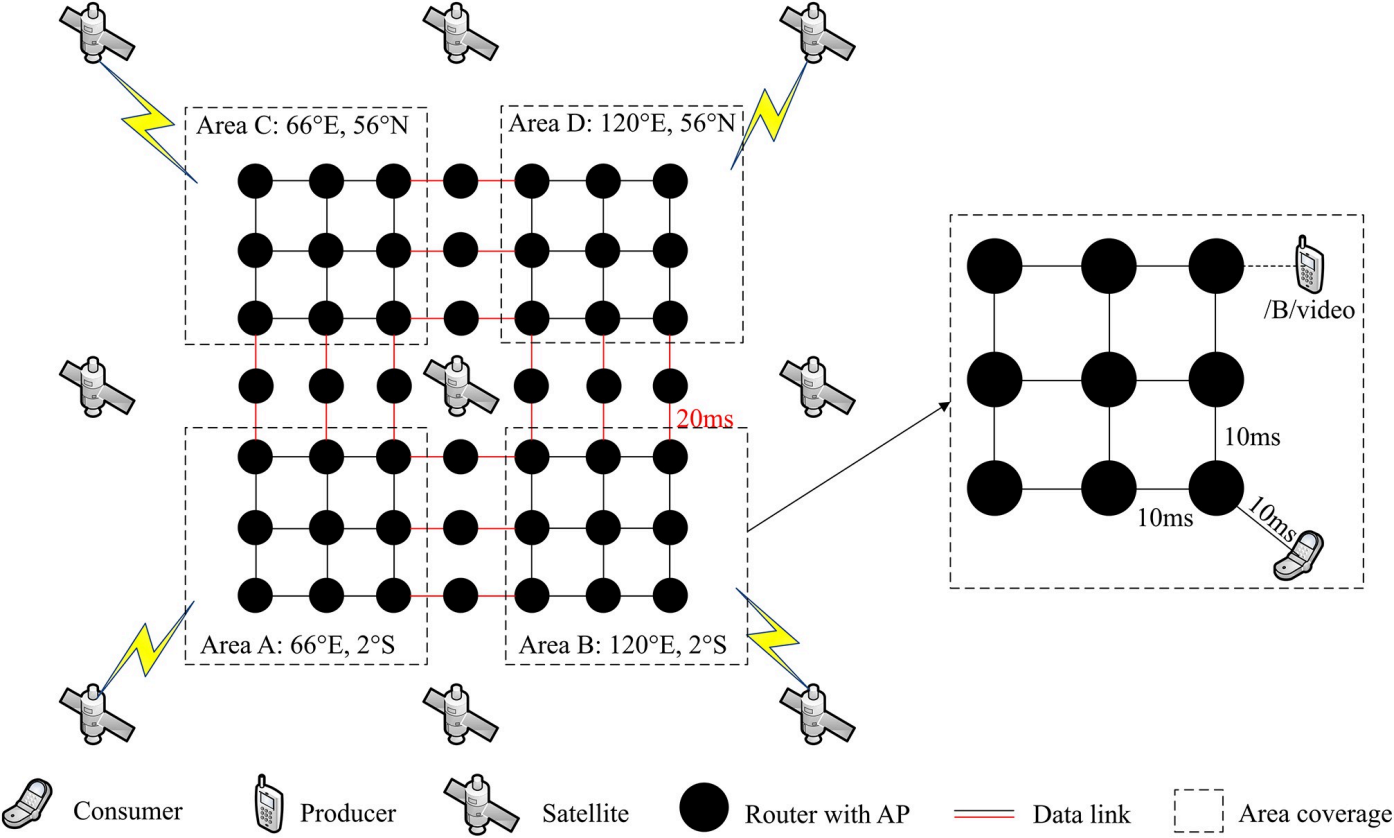
The diagram of simulation topology.

To evaluate the performance of MsDD, we conducted simulation experiments using ndnSIM, a simulation software specifically designed for NDN based on ns3 [33]. We built the required experimental environment according to the network model of MsDD, which consists of an Iridium constellation, 3 GEO satellites, and ground-layer equipment. The topology of the ground-layer equipment is shown in Fig 8 and is composed of 4 equally sized interconnected network areas, each covered by a different LEO satellite. Moreover, each network area has a stationary consumer and a mobile producer within that area, both of which can communicate directly with the LEO satellites. To simulate a realistic environment, we selected four suitable global locations to place the 4 network areas and used the Satellite Tool Kit (STK) to simulate the dynamics of the Iridium constellation. Specific parameters are listed in Table 1.

**Table 1:** Simulation parameters.

	Parameter	Default value	Range
General	Simulation duration	100s	
	Number of LEO	66	
	Number of orbits	6	
	Number of GEO	3	
Application	Request rate	30req/s	10–50req/s
	Request pattern	Zipf, *α* = 1	
	Data packet size	100KB	
Mobility	Speed	20m/s	10–30m/s
	Model	RandomWalk2dMobilityModel	
Cache	Cache size	100objects	
	Cache replacement	LRU	
MsDD	Number of managers	1,2,3,4,11	
	*ε*	0.5	
	*τ*	10s	
Ground topology	Routers with AP	49	
	Per area size	1200 × 1200m	
	AP range	200m	
	Router’s links	100Mbps	
	Handoff delay(layer 2)	200ms	

### 4.2 Schemes and evaluation metrics

To evaluate the performance of MsDD, we initially need to conduct experiments by varying the number of managers in each orbit within the LEO layer to evaluate the impact of the number of managers on the performance of MsDD. Subsequently, we will verify the advantages of the proposed in-network caching scheme in MsDD. Finally, we compare MsDD with other mobility support schemes on three main indicators that reflect consumer satisfaction [34]: (1) Consumer delay: Calculated as the time difference between the initial attempt to send an Interest and the successful reception of the data at the consumer. This metric is crucial as it measures the responsiveness of the system from the consumer’s perspective. (2) Delivery Ratio: Represents the proportion of successfully received data packets to the total number of Interest packets sent by the consumer. This ratio indicates the efficiency of the system in delivering data to the consumer. (3) Signaling Overhead: The number of messages required to ensure that the consumer’s Interest packets can reach the mobile producer during a handover event. This metric reflects the overhead imposed on the network to maintain continuous communication as the producer moves.

### 4.3 Simulation result

**Fig 9:**
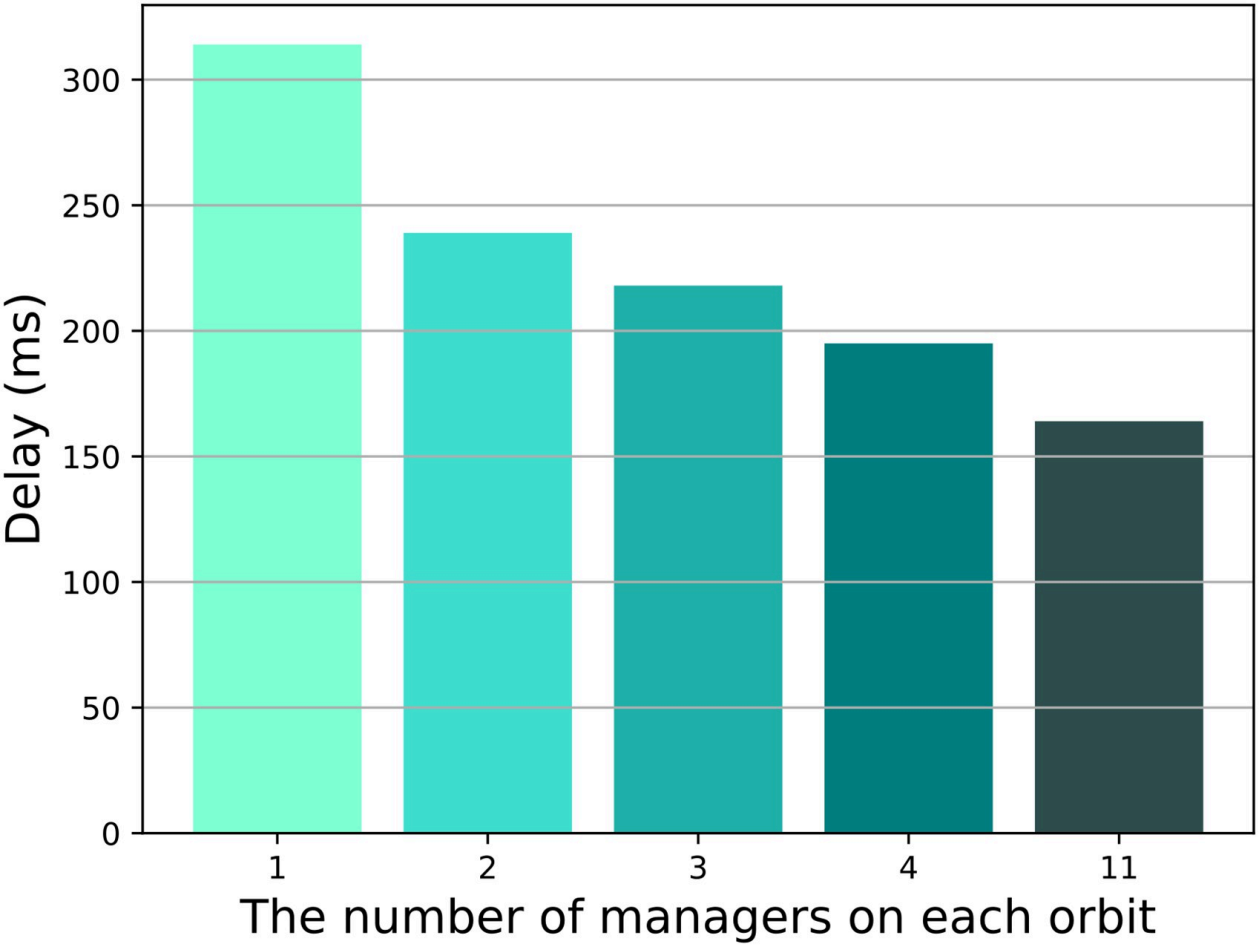
Impact of the number of managers on consumer delay.

The first scenario evaluates the impact of the number of managers on the performance of MsDD. The results depicted in Fig 9 indicate a clear downward trend in consumer delay as the number of managers increases. This is attributed to the fact that the more managers on a orbit, the fewer additional hops the interest and data packets need to forward to their nearest manager. When the number of managers on each orbit reaches 11, the additional hops become 0, hence the consumer delay is at its minimum.

**Fig 10:**
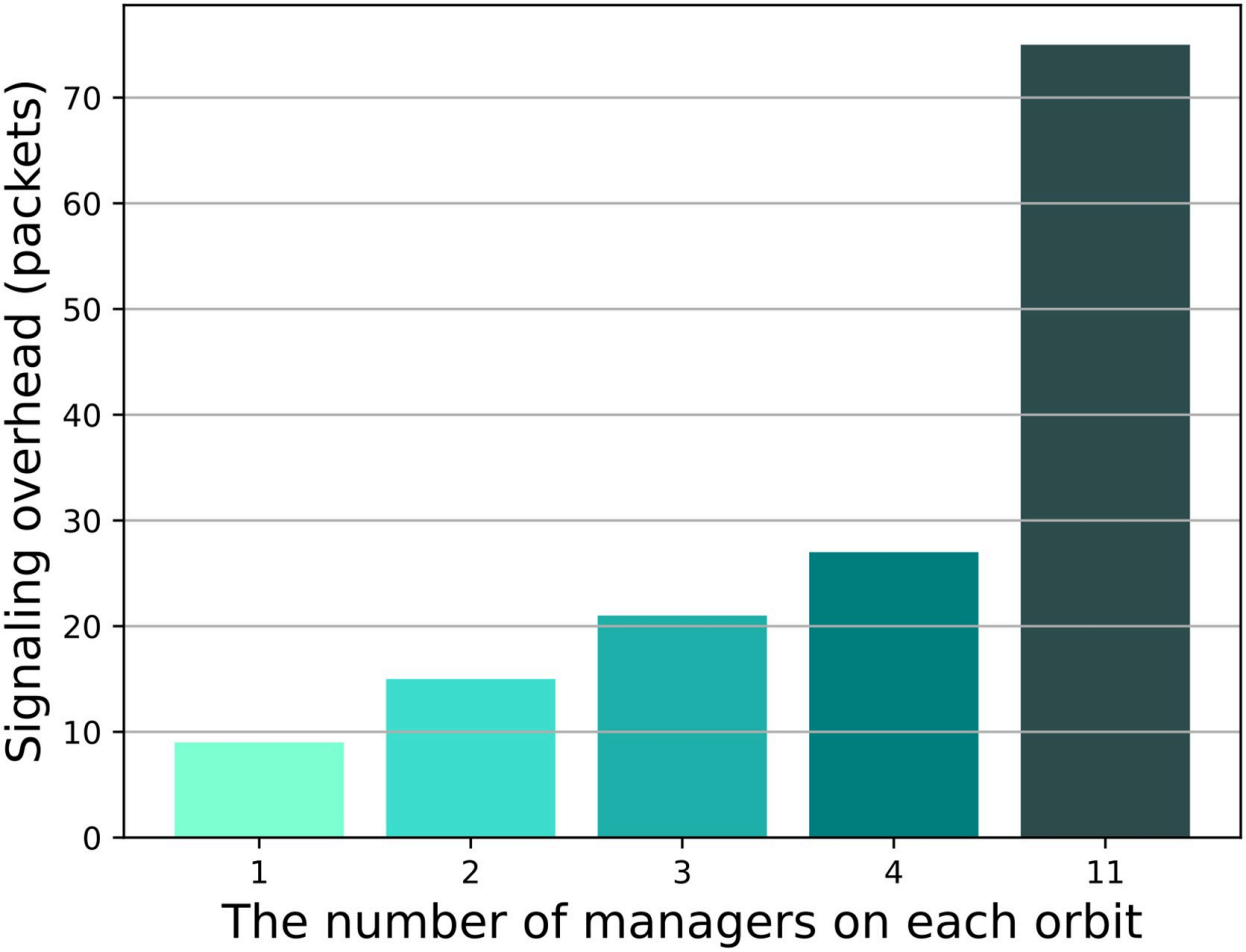
Impact of the number of managers on signaling overhead.

The results from Fig 10 indicate that as the number of managers increases, the signaling overhead for each update of the DL during the data collection phase also becomes higher. This is because during the data collection phase, managers need to send update information to the GEO satellite, and as the number of managers increases, more managers will be sent update information from the GEO satellites. Comparing with Fig 9, we can see that when there is 1 manager per orbit, although the signaling overhead is minimal, it results in a larger consumer delay. Conversely, when there are 11 managers per orbit, although the consumer delay is reduced, it leads to a significant signaling overhead. Therefore, we believe that placing 1 or 11 managers per orbit does not optimize the performance of MsDD, so in subsequent experimental processes, we have excluded these two schemes.

**Fig 11:**
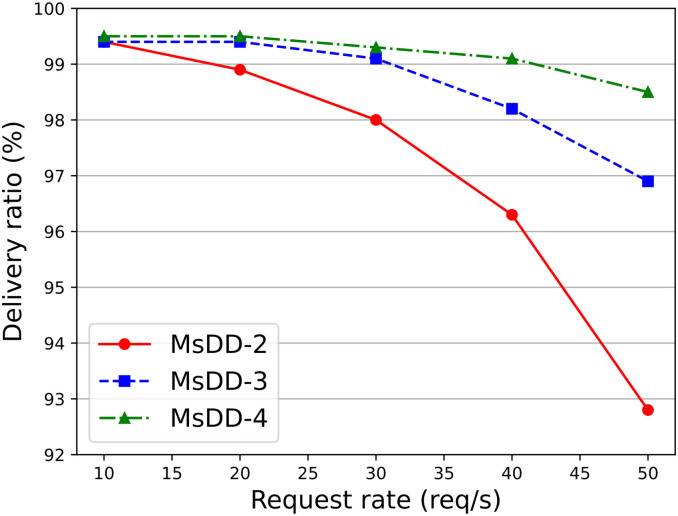
Impact of the number of managers on delivery ratio as request rate increases.

We have named the schemes with 2, 3, and 4 managers per orbit as MsDD-2, MsDD-3, and MsDD-4, and compared their delivery ratios as the request rate increases. Fig 11 shows that when the request rate increases, the delivery ratio of all three schemes tends to decline due to the increase in data volume within the network, with MsDD-2 and MsDD-3 showing a more pronounced decrease. This is because in MsDD, the manager nodes carry a large amount of network traffic, and the fewer managers there are, the greater the load on each manager and the inter-satellite links between different orbits. Therefore, we believe that when there are 2 or 3 managers per orbit, the performance of MsDD is also not optimal, so in subsequent experimental processes, we have excluded the schemes MsDD-2 and MsDD-3 and selected MsDD-4 for further experiments.

**Fig 12:**
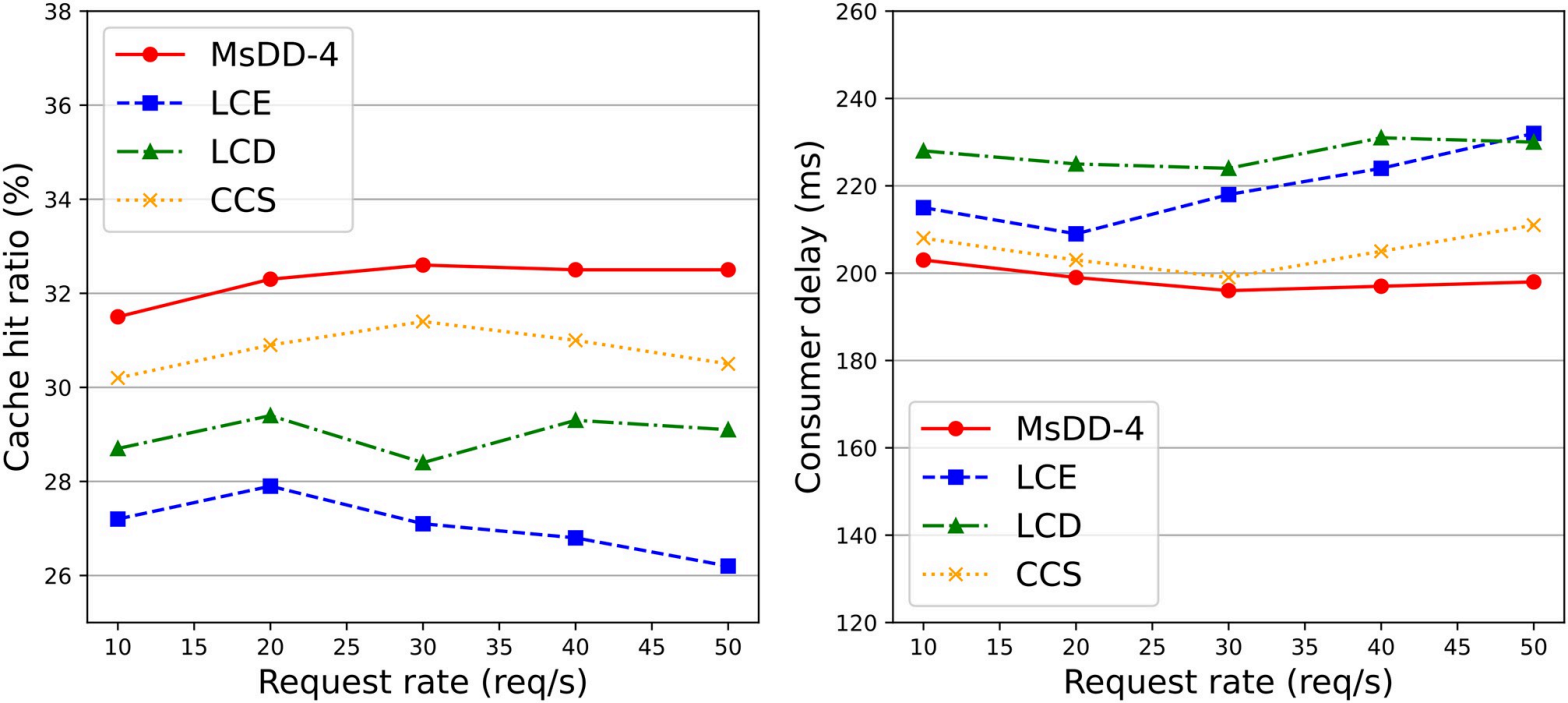
Performance changes of different in-network caching strategies as request rate increases. The left figure shows the change in cache hit rate as the request rate increases, and the right figure shows the change in consumer delay as the request rate increases.

The second scenario assesses how in-network caching strategy of MsDD-4 performs compared to LCE [24], LCD [25], and CCS [29] in terms of cache hit rate and consumer delay when the request rate changes, as depicted in Fig 12. From Fig 12, it can be seen that as the request rate increases, the cache hit rate and consumer delay of MsDD-4 are significantly better than other schemes. This is because in-network caching strategy of MsDD-4 prioritizes caching more popular content in the network, reducing cache redundancy and also reducing the probability of requested content being replaced. At the same time, MsDD-4 increases the probability of data packets cached near the manager, allowing interest packets to hit the cache with fewer hops.

The final scenario evaluates the performance of MsDD-4 when the producer’s mobility speed changes, comparing it with several other NDN producer mobility support schemes. We selected the inherent forwarding strategy of NDN (Pure NDN), two popular schemes from the references (KITE [13] and MAP-ME [19]), and a caching-based scheme from the references (T-Move [22]) for comparative analysis.

**Fig 13:**
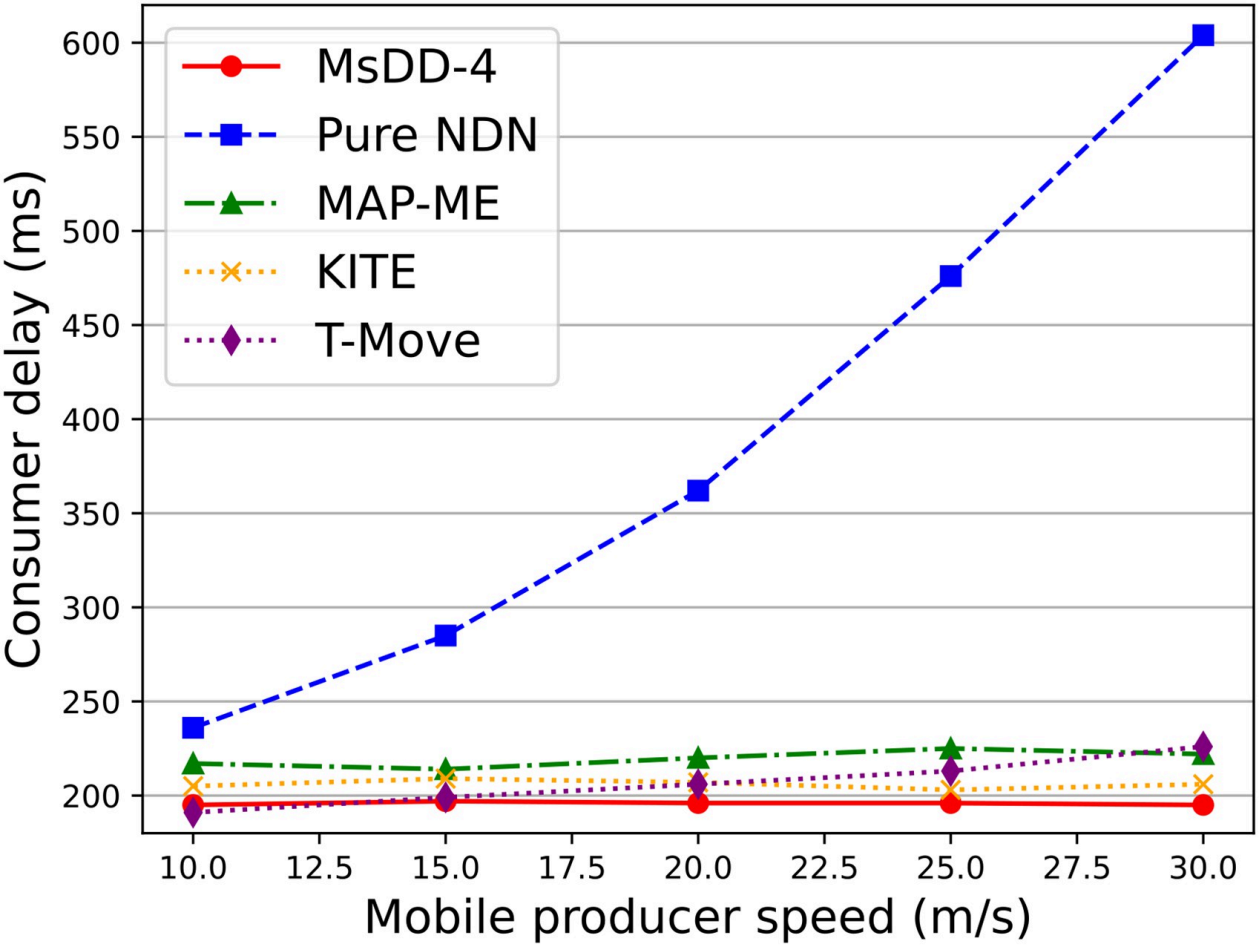
Impact of the producer mobility speed on consumer delay.

We first evaluate the changes in consumer delay for these mobility support schemes when the producer mobility speed changes. The results in Fig 13 indicate that with the increase of producer mobility speed, except for Pure NDN, all other schemes have good performance in consumer delay and remain stable within a reasonable numerical range. And MsDD-4 performs the most stably, and increases consumer delay by about 5% compared to other solutions. The main reasons are threefold:

- MsDD converges consumers and data packets on low Earth orbit satellites, so the consumer delay of MsDD is only affected by the satellite network, not the terrestrial network.
- Apart from satellite handover, MsDD does not have other handoff latency, whether it is layer 2 latency or mobility management latency, which greatly reduces the consumer delay of MsDD.
- Due to the superior cache hit rate of MsDD’s in-network caching strategy within the data depot, this allows data packets to be obtained with fewer hops, reducing consumer delay.

**Fig 14:**
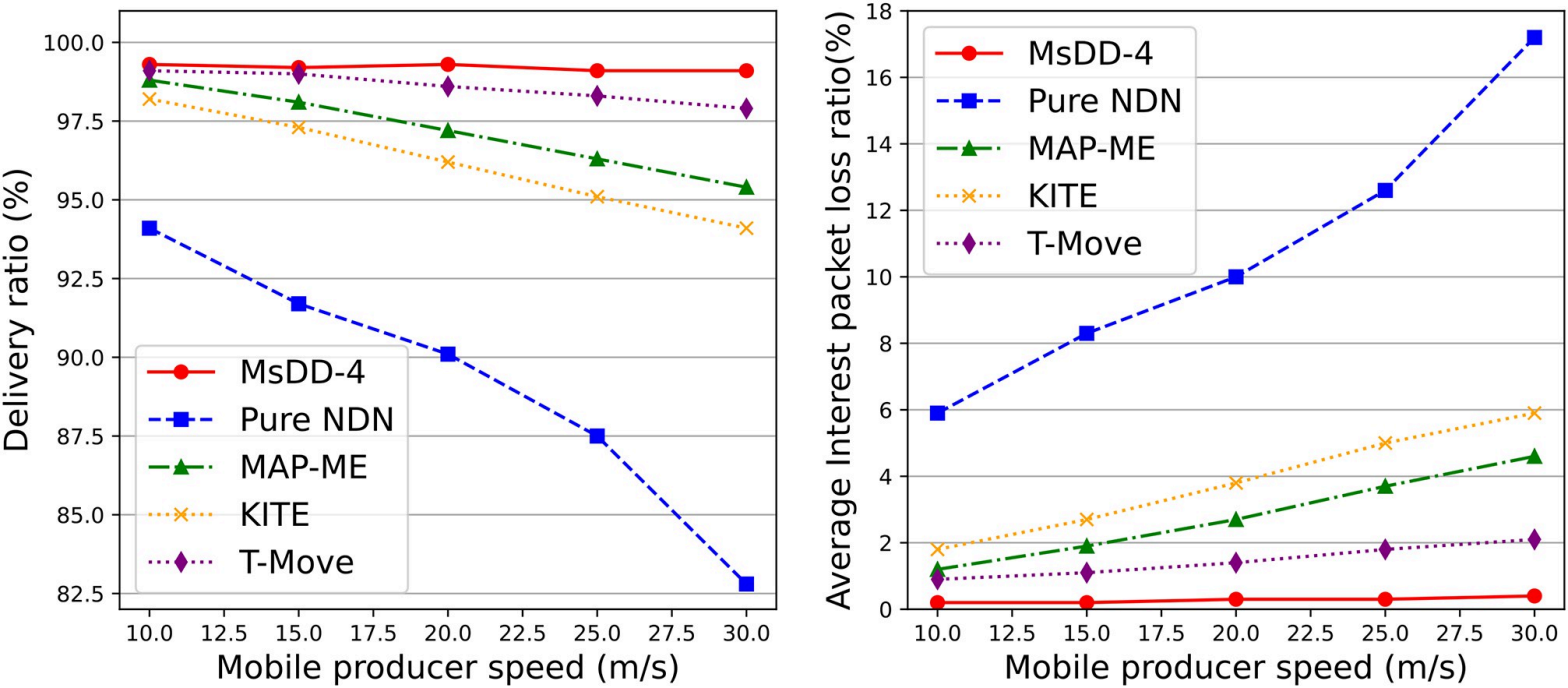
Impact of the producer mobility speed on delivery ratio and average interest packet loss ratio. The left figure shows the change in delivery ratio as the producer mobility speed increases, and the right figure shows the change in average interest packet loss ratio as the producer mobility speed increases.

The results from Fig 14 demonstrate that the delivery ratio and average interest packet loss ratio of MsDD-4 is significantly superior to other schemes, and this advantage becomes more pronounced as the producer’s mobility speed increases. This is because while other schemes can mitigate packet loss during handover to a certain extent, an increase in handover events inevitably leads to a decrease in the delivery ratio. For instance, with KITE, as the producer’s mobility speed increases, the frequency of switches between APs also rises, leading to stale path issues and resulting in packet loss. However, in MsDD, packet loss typically occurs during satellite handover and inter-satellite link switching, the former being a low-probability and acceptable event, and for the latter, our proposed routing strategy and reverse seam strategy provide effective solutions.

**Fig 15:**
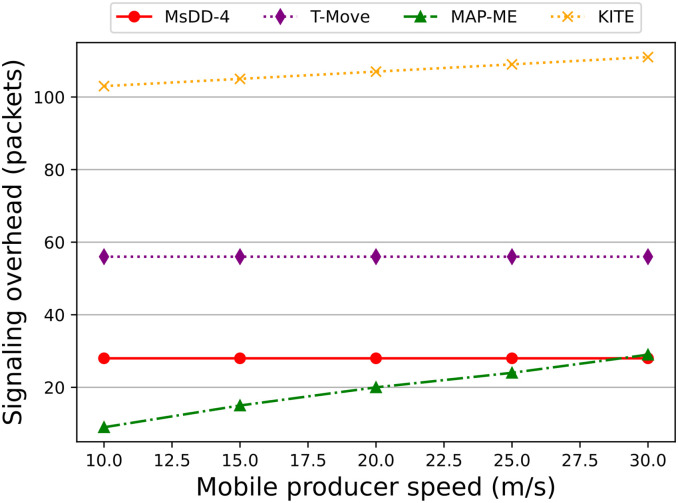
Impact of the producer mobility speed on signaling overhead.

Fig 15 illustrates the changes in signaling overhead as the producer mobility speed increases, where we calculate the signaling overhead of MsDD during satellite handover. It can be observed that the signaling overhead of MsDD remains stable with changes in the producer’s mobility speed and is superior to that of KITE and T-Move. This is because when the producer moves, KITE needs to frequently send TI/TD packets to the producer to update the tracking path, while T-Move requires sending messages to update the FIB before and after the handover. In contrast, the signaling overhead of MsDD is only related to the number of managers, as the GEO controller only sends update information to the managers.

## 5 Conclusion and future work

To address the seamless communication issue in NDN under producer mobility, we have proposed MsDD. MsDD leverages the characteristics of a LEO satellites constellation, procaching the data packets generated by producers onto LEO satellites, and guiding the interest packets to retrieve data through satellites. We design the basic model of the data depot, a routing strategy based on forwarding hint, and a probability-based in-network caching strategy to support our approach, and validated the rationality and advantages of our proposed scheme through simulation. The simulation results show that, compared to several other mobility support schemes, MsDD maintains stable consumer delay, delivery ratio, and signaling overhead under the environment of frequent producer mobility, and the consumer delay and delivery ratio are superior to other schemes in the scenarios we proposed. The simulation results demonstrate that MsDD can effectively shield the impact of frequent producer mobility on network performance.

Our future work includes: (1) further improving the feasibility and practical relevance of MsDD in more real-world scenarios, such as desert and marine environments. (2) researching the impact of satellite capacity and energy consumption on MsDD as network traffic increases. (3) resolving consumer mobility issues caused by the dynamic nature of LEO constellations in MsDD.

## Supporting information

S1 File Experimental data. (PDF)
