# Supplementary material for: MsDD: A novel NDN producer mobility support scheme based on multi-satellite data depot
Source: PLoS One. 2024 Sep 12;19(9):e0310379. doi: 10.1371/journal.pone.0310379 (PMC11392351; doi:10.1371/journal.pone.0310379)
Supplement: S1 File — (PDF) [file pone.0310379.s001.pdf]

Fig	Subfigure	Data Points				
fig 9		314	239	218	195	164
fig 10		9	15	21	27	75
fig 11		99.4	99.4	99.1	98.2	96.9
		99.4	98.9	98.0	96.3	92.8
		99.5	99.5	99.3	99.1	98.5
fig 12	left	31.5	32.3	32.6	32.5	32.5
		27.2	27.9	27.1	26.8	26.2
		28.7	29.4	28.4	29.3	29.1
		30.2	30.9	31.4	31.0	30.5
	right	203	199	196	197	198
		215	209	218	224	232
		228	225	224	231	230
		208	203	199	205	211
fig 13		195	197	196	196	195
		236	285	362	476	604
		217	214	220	225	222
		205	209	207	203	206
		191	199	206	213	226
fig 14	left	99.3	99.2	99.3	99.1	99.1
		94.1	91.7	90.1	87.5	82.8
		98.8	98.1	97.2	96.3	95.4
		98.2	97.3	96.2	95.1	94.1
		99.1	99.0	98.6	98.3	97.9
	right	0.22	0.21	0.28	0.33	0.40
		5.91	8.32	9.92	12.63	17.15
		1.20	1.89	2.73	3.66	4.61
		1.81	2.72	3.79	5.02	5.93
		0.90	1.11	1.42	1.84	2.08
fig 15		28	28	28	28	28
		9	15	20	24	29
		103	105	107	109	111
		56	56	56	56	56
